# Fatty Kidney: The Interplay of Lipids and Diabetic Kidney Disease

**DOI:** 10.3390/biomedicines14040944

**Published:** 2026-04-21

**Authors:** Zhiyue Zou, Pan Gao, Qian Yuan, Zhiwen Wang, Pengli Luo, Chun Zhang

**Affiliations:** 1 Department of Nephrology, Union Hospital, Tongji Medical College, Huazhong University of Science and Technology, Wuhan 430022, China; zouzhiyue@hust.edu.cn (Z.Z.); panpangao1121@163.com (P.G.); qianyuan@hust.edu.cn (Q.Y.); wangzhiwenncu@163.com (Z.W.); 2 Department of Nephrology, Affiliated Hospital of Qinghai University, Xining 810001, China

**Keywords:** diabetic nephropathy, diabetic atherosclerosis, oxidative stress, lipotoxicity

## Abstract

Diabetic nephropathy and diabetic atherosclerosis often develop together and share similar metabolic disturbances. Lipid abnormalities are common in diabetes, yet their roles in kidney and vascular injury are not fully understood. In diabetic kidney disease, altered lipid uptake, reduced fatty acid oxidation, and accumulation of harmful lipid species contribute to cellular stress, mitochondrial injury, inflammation, and fibrosis. In parallel, disordered lipid handling in the vasculature promotes endothelial dysfunction and atherosclerotic plaque development. However, not all lipid accumulation appears to be detrimental, and some findings suggest adaptive or context-dependent effects, leading to inconsistent results across studies. In this review, we summarize current evidence on lipid metabolism in diabetic nephropathy and atherosclerosis, compare shared and distinct features, and discuss ongoing controversies. We also briefly address the therapeutic relevance of targeting lipid pathways and highlight areas that require further investigation. Compared with prior reviews that mainly discussed fatty kidney as an emerging concept in chronic kidney disease research, this review specifically focuses on diabetic kidney disease and integrates kidney-specific lipid trafficking, kidney–vessel crosstalk, conflicting evidence, and mechanism-based therapeutic implications.

## 1. Introduction

Diabetic kidney disease (DKD) is a major driver of chronic kidney disease (CKD) progression and remains a leading cause of kidney failure worldwide [1,2]. In patients with diabetes, cardiovascular disease accounts for most deaths and is largely attributable to accelerated atherosclerosis and metabolic dysfunction [3,4]. This risk is further amplified in the presence of chronic kidney disease, where cardiovascular morbidity and mortality are substantially elevated [5,6]. Reduced estimated glomerular filtration rate (eGFR) and albuminuria independently predict cardiovascular events and death, further highlighting the close interrelationship between renal and vascular injury [6,7]. Together, these findings suggest that kidney and vascular injury share overlapping pathophysiological mechanisms rather than representing isolated complications [3,8].

Among the proposed mechanisms, disordered lipid metabolism represents one potential shared pathway [8,9]. Dyslipidemia, characterized by elevated triglycerides, qualitative changes in low-density lipoproteins, and reduced high-density lipoprotein cholesterol, plays a central role in the development of diabetic macrovascular disease [3,9]. Increasing evidence also indicates that lipid handling within renal cells is altered in diabetes and chronic kidney disease, promoting oxidative stress, inflammation, and fibrotic remodeling [10]. This recognition of kidney lipid dysmetabolism and lipid droplet accumulation provides a mechanistic framework linking metabolic stress to structural kidney damage [10,11,12,13].

**Figure 1:**
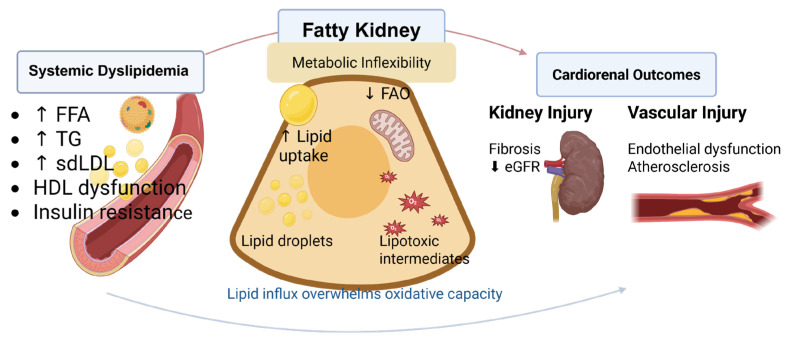
Conceptual framework linking systemic dyslipidemia to fatty kidney and cardiorenal outcomes. Diabetic dyslipidemia, characterized by elevated circulating free fatty acids (FFAs), triglycerides (TGs), small dense low-density lipoproteins (sdLDL), and dysfunctional high-density lipoproteins (HDL), increases lipid flux to peripheral tissues, including the kidney. In proximal tubular epithelial cells, enhanced lipid uptake (e.g., via CD36/FATP2) combined with impaired mitochondrial fatty acid oxidation (FAO) promotes lipid droplet accumulation and generation of lipotoxic intermediates, reflecting renal metabolic inflexibility that contributes to the fatty kidney phenotype. When lipid influx exceeds oxidative capacity, these processes drive cellular stress, inflammation, and fibrotic remodeling, contributing to progressive decline in kidney function. In parallel, systemic and renal lipid dysregulation promotes endothelial dysfunction and atherosclerosis, leading to adverse cardiovascular outcomes. Kidney and vascular injury are linked through bidirectional cardio–renal–metabolic crosstalk, forming a self-reinforcing pathogenic loop. Created in BioRender. Zhang, C. (2026) https://BioRender.com/c2vvj8c (accessed on 9 March 2016).

Importantly, lipid accumulation per se does not necessarily equate to toxicity. Lipid droplet formation may serve an adaptive function under conditions of transient lipid excess, whereas sustained lipid overload reflects metabolic imbalance and contributes to progressive organ injury [10]. Whether lipid accumulation represents adaptive buffering or maladaptive lipotoxicity remains incompletely understood. Clarifying this distinction is critical for interpreting experimental findings and for identifying effective therapeutic targets in DKD and cardiometabolic disease [8,10]. In this context, the concept of the “fatty kidney” may provide a useful integrative framework. In the literature, fatty kidney generally refers to ectopic lipid accumulation in and around the kidney, including the renal parenchyma, glomerular and tubular compartments, and, in a broader anatomic sense, the renal sinus/hilum and perirenal region [10,11,12,13]. In this review, we use the term primarily to describe this renal lipid deposition phenotype in diabetic kidney disease. We further discuss a related functional dimension, namely renal metabolic flexibility, which refers to the ability of renal cells to adapt substrate utilization and maintain lipid homeostasis according to metabolic demand, whereas renal metabolic inflexibility refers to failure of this adaptive response under metabolic stress. In practical terms, this includes increased lipid influx and uptake, altered glomerular lipid filtration and tubular reabsorption, impaired mitochondrial fatty acid oxidation, maladaptive lipid droplet storage, and insufficient lipid disposal or efflux. Accordingly, renal metabolic inflexibility should be considered a mechanistic contributor to, rather than a synonym for, the fatty kidney phenotype. This distinction may help reconcile conflicting findings and guide more precise investigation of lipid-targeted interventions (Figure 1). In contrast to prior reviews that mainly introduced fatty kidney as a future direction in CKD research, the present review emphasizes fatty kidney as an integrative framework for diabetic kidney disease, with particular attention to renal lipid handling, vascular crosstalk, conflicting evidence regarding adaptive versus maladaptive lipid storage, and translational therapeutic opportunities.

### Literature Search Strategy

For this narrative review, we searched PubMed, Web of Science, and Scopus for relevant articles published up to February 2026. Search terms included combinations of “fatty kidney”, “diabetic kidney disease”, “diabetic nephropathy”, “lipid metabolism”, “lipotoxicity”, “fatty acid oxidation”, “proximal tubule”, “atherosclerosis”, “PCSK9”, “SGLT2 inhibitors”, and related keywords. We prioritized English-language original studies, mechanistic reports, clinical trials, and high-quality review articles relevant to renal lipid dysmetabolism, vascular injury, and kidney–vessel crosstalk. Reference lists of selected articles were also screened manually to identify additional relevant studies.

## 2. Lipid Metabolism in Health

### 2.1. Physiological Roles

Lipids are essential macromolecules that shape membrane composition and organization, provide high-energy substrates, and generate signaling intermediates, thereby linking cellular structure to inflammation, apoptosis, and metabolic adaptation [8,10]. Crucially, lipid species and their metabolites can shift from physiological mediators to stress signals when present in excess, and lipid accumulation is therefore increasingly viewed as a mechanistic contributor to tissue inflammation and fibrosis rather than a passive bystander [10,14,15].

In the diabetic milieu, dysregulated lipid flux is frequently coupled to oxidative stress and organelle dysfunction, providing a coherent framework for how chronic nutrient surplus is transduced into cellular injury programs [8,14,16]. Conceptually, lipid homeostasis is best framed as a regulated balance among uptake, de novo synthesis, mitochondrial oxidation, storage in lipid droplets, and efflux, which together maintain functional lipid pools while preventing lipotoxicity [10,11,12]. Within this broader landscape, lipid-derived mediators can also participate in inflammation resolution and restoration of tissue function, underscoring that lipid biology includes both pathogenic and pro-homeostatic programs [13,17].

Taken together, this perspective is clinically actionable because multiple nodes in lipid handling and mitochondrial fitness are pharmacologically targetable, motivating therapeutic strategies that intervene upstream of irreversible scarring [16,18,19].

### 2.2. Vascular Lipid Handling

The vasculature is continuously exposed to circulating lipoproteins and inflammatory cues, making tight regulation of intracellular cholesterol burden central to preserving endothelial and immune-cell function [6,20,21].

ABCA1-mediated cholesterol efflux to ApoA-I and nascent HDL constitutes a key protective checkpoint that limits foam-cell formation and restrains vascular inflammation [21]. Consistent with this mechanism, HDL is increasingly interpreted through functional properties—such as efflux capacity and anti-inflammatory activity—rather than HDL-C concentration alone [22].

In diabetes and CKD, systemic dyslipidemia and chronic inflammation converge to accelerate atherosclerotic processes, accounting for the disproportionate cardiovascular event burden highlighted in contemporary epidemiology and guidance documents [3,6,20].

Accordingly, major professional guidelines prioritize integrated cardiometabolic risk reduction (including lipid lowering) in diabetes, with special attention to patients with CKD who carry amplified risk [3,4,23]. In this context, vascular lipid handling is better viewed as a parallel driver of outcomes—not merely a comorbidity—within metabolic kidney disease [20,24].

### 2.3. Renal Lipid Handling

The kidney—particularly proximal tubule epithelium—has exceptional ATP requirements and relies heavily on mitochondrial FAO, making tubular cells uniquely vulnerable to perturbations in mitochondrial homeostasis [19,25]. Across CKD, lipid droplet accumulation and broader lipid dysmetabolism are increasingly recognized as common correlates of tubular injury, inflammation, and progressive fibrosis [10,13]. Mechanistically, mitochondrial oxidative damage can directly reprogram tubular lipid metabolism in diabetic kidneys, linking energy failure to sustained profibrotic signaling [26,27]. Complementary syntheses emphasize that lipotoxicity in kidney disease often reflects a mismatch between lipid delivery and oxidative capacity, which amplifies oxidative stress and cell-death pathways that drive tubulointerstitial remodeling [14,15]. Causal support for the protective role of FAO comes from tubular carnitine palmitoyltransferase 1A (CPT1A) gain-of-function, which restores mitochondrial homeostasis and attenuates kidney fibrosis in vivo [25,28].

Renal lipid metabolism also differs from systemic lipid metabolism in several important respects. Unlike tissues in which lipid exposure primarily reflects circulating substrate availability, the kidney is uniquely influenced by glomerular filtration and tubular reabsorption [10,14,15,19]. Proximal tubular epithelial cells have limited glycolytic reserve and depend predominantly on mitochondrial fatty acid oxidation to meet their high ATP demand, making them especially vulnerable to lipid overload when oxidative capacity is impaired [19,25,26,27]. In the setting of glomerular barrier injury, albumin-bound fatty acids, lipoprotein-derived lipids, and other filtered lipid cargo may be delivered in excess to the proximal tubule, where receptor- and transporter-mediated uptake pathways further amplify intracellular lipid loading [29,30,31,32,33,34]. This combination of high lipid exposure and high oxidative dependence makes the kidney especially vulnerable to ectopic lipid accumulation when uptake, synthesis, storage, oxidation, and efflux become imbalanced [10,13,14,15,19]. In addition to neutral triglycerides stored in lipid droplets, the renal lipid milieu includes fatty acids, phospholipids, sphingolipids, cholesteryl esters, and bioactive intermediates such as ceramides and lysophosphatidylcholine, which may exert distinct biological effects depending on cell type and subcellular localization [10,35,36,37].

At the pathway level, kidney cell-type lipid handling involves coordinated control over uptake, oxidation, storage, and efflux, and disruption of this coordination is now positioned as a central feature of disease progression models [10,19]. Single-cell and multimodal profiling further indicates that DKD progression involves coordinated shifts in transcriptional programs and chromatin accessibility that intersect metabolic and inflammatory pathways, including proximal tubule injury states [38,39]. Related single-cell atlases, including therapy-response studies, support partial reversibility of these disease-associated states, strengthening the inference that metabolic programs are tractable determinants rather than purely downstream markers [38,40]. Increased tubular lipid uptake represents one route to lipid overload, exemplified by Fatty Acid Transport Protein 2 (FATP2)-linked programs that promote tubular lipid metabolic reprogramming and are associated with DKD progression [41,42]. Beyond uptake, pharmacologic inhibition of FABP4 can reprogram tubular lipid metabolism and attenuate fibrotic remodeling in experimental systems, supporting flux redirection as a therapeutic concept [18,43]. At the level of lipid droplet turnover, ATGL-dependent remodeling of lipid metabolism influences fibrosis during the AKI-to-CKD transition [44].

In parallel, glycolysis–lipid metabolism crosstalk can promote renal lipid loading and fibrosis, as illustrated by an IGFBP7–PKM2 axis in tubular epithelial cells [45]. Immune–metabolic interactions add another control layer, with Trem2^+^ macrophages reported to alleviate tubular lipid accumulation and ferroptosis in diabetic nephropathy models [46]. Glomerular lipid handling is likewise implicated, with Proprotein Convertase Subtilisin/Kexin Type 9 (PCSK9) linked to glomerular lipid accumulation and renal injury, and CCDC92-associated pathways influencing podocyte lipotoxicity in DKD contexts [29,30]. Finally, podocyte–tubule communication can shape metabolic injury states, as podocyte-specific KLF6 primes proximal tubule signaling programs that attenuate DKD, underscoring nephron-wide integration of lipid/energy stress [47].

Taken together, renal lipid handling in health reflects coordinated control of lipid flux and mitochondrial capacity, and its breakdown in diabetes/CKD motivates combined mitochondrial and lipid-metabolic targeting strategies already being advanced in translational pharmacology and consensus guidance [1,10,16,18,24].

## 3. Lipid Dysregulation in Diabetic Kidney Disease

### 3.1. Systemic Dyslipidemia

DKD unfolds against the background of systemic metabolic dysfunction. Within this context, diabetic dyslipidemia is increasingly appreciated as more than a cardiovascular comorbidity, because it reshapes the lipid milieu delivered to the kidney and can influence cellular stress responses once renal handling capacity is exceeded [10]. In insulin-resistant diabetes, impaired insulin-mediated suppression of adipose lipolysis increases circulating non-esterified fatty acids, whereas hepatic “selective” insulin resistance favors dysregulated hepatic lipid handling and VLDL overproduction, contributing to hypertriglyceridemia [3,48,49,50,51]. Clinically, type 2 diabetes is marked not only by elevated triglycerides and reduced HDL-C, but also by a shift toward a more atherogenic apoB-lipoprotein profile, including a higher burden of small, dense LDL particles that tend to persist longer in the circulation and are more prone to oxidative or glycation-related modification under diabetic conditions [48,49,52]. Taken together, these abnormalities increase lipid flux to peripheral tissues; in the kidney, sustained exposure to fatty acids, triglyceride-rich lipoproteins/remnants, and modified lipoproteins aligns with a model in which metabolic overload amplifies inflammatory and profibrotic programs, particularly in tubular compartments that sit at the intersection of high energy demand and lipid substrate supply [10,50].

### 3.2. Renal Lipid Accumulation

A convergent set of human and experimental data supports ectopic renal lipid deposition as a reproducible feature of DKD, with lipid droplets and dysregulated lipid species reported across glomerular and tubular compartments [10]. Importantly, this pattern is more consistent with coordinated changes in uptake, utilization, and storage than with passive “spillover” alone. Proximal tubules are especially relevant here: they operate near a high energetic threshold and depend heavily on mitochondrial fatty acid oxidation, so disruptions in mitochondrial programs can reduce lipid disposal capacity and predispose to intracellular lipid sequestration [19,53,54].

A key kidney-specific feature of fatty kidney pathogenesis is the coupling of glomerular lipid filtration to tubular lipid reabsorption. In diabetes, glomerular barrier dysfunction may increase the delivery of albumin-bound fatty acids and other lipid-containing macromolecules to the tubular lumen [10,11,12,29,30]. Proximal tubular cells then reabsorb these filtered lipids through receptor- and transporter-mediated pathways, including Kidney Injury Molecule-1 (KIM-1)- and FATP2-associated mechanisms [31,32,34,42]. When filtered lipid load persistently exceeds mitochondrial oxidative capacity, excess lipids are redirected toward esterification and lipid droplet storage, while bioactive lipid intermediates accumulate [10,14,33]. This mismatch between lipid delivery and disposal provides a mechanistic bridge between systemic dyslipidemia and ectopic renal lipid deposition [10,12,19].

Under chronic metabolic stress, this excess lipid burden is not confined to a benign storage pool. Rather, lipid spillover into the renal parenchyma, together with oxidative stress and mitochondrial dysfunction, promotes inflammatory signaling, tubular injury, endothelial activation, and progressive fibrotic remodeling [11,12,53,54,55,56]. Thus, the fatty kidney phenotype should be understood not simply as lipid deposition, but as a dynamic process in which filtered and reabsorbed lipid cargo, maladaptive intracellular handling, and parenchymal injury reinforce one another [10,19,55].

Mechanistically, recent work has highlighted active lipid uptake routes in tubular epithelium, including KIM-1-mediated uptake of fatty acid-bound albumin that aggravates tubulointerstitial inflammation and fibrosis, directly linking tubular lipid handling to DKD progression [31,32]. In parallel, FAO insufficiency—whether through broader mitochondrial dysfunction or specific metabolic constraints—promotes intracellular lipid accumulation that associates with worse kidney function, as shown in recent human and experimental DKD studies [33,53]. Complementing these pathways, altered tubular fatty-acid transport/activation has been implicated via FATP2-dependent mechanisms, and genetic or pharmacologic perturbation of FATP2 has been shown to modulate DKD phenotypes in preclinical and translational studies [34,42].

Beyond neutral lipids, kidney lipidomic signals also nominate bioactive lipid species as potential progression correlates; for example, lysophosphatidylcholine dysregulation has been linked to “fast decliner” trajectories in DKD cohorts and experimental models, supporting the concept that specific lipid species may mark (and potentially mediate) aggressive disease states [36]. Collectively, the field increasingly supports a model in which renal lipid accumulation tracks with fibrosis and functional loss, while the degree to which lipid deposition is causal versus a marker of broader metabolic decompensation remains an active—and clinically relevant—question [10,19].

These kidney-specific abnormalities in lipid trafficking provide the upstream basis for the oxidative stress, mitochondrial injury, inflammatory activation, and endothelial dysfunction discussed in the following sections.

### 3.3. Lipid Deposition and Diabetic Vascular Injury

The renal lipid phenotype parallels key features of diabetic vascular injury, where hyperglycemia and dyslipidemia reinforce each other to amplify oxidative stress, endothelial activation, and lipid retention within vessel walls [56,57]. Contemporary cardiovascular/diabetes-focused reviews emphasize that diabetic dyslipidemia increases atherogenic lipoprotein burden and promotes a pro-oxidant environment conducive to lipoprotein modification and inflammatory signaling [49,56,57,58]. At the cellular level, endothelial dysfunction remains a pivotal early event, characterized by impaired nitric oxide signaling and heightened inflammatory responses, thereby facilitating leukocyte recruitment and progression of atherosclerotic lesions [56,57,58]. In turn, modern atherosclerosis syntheses highlight foam-cell biology as a central pathologic axis driven by altered lipid handling and inflammation, linking modified lipoproteins to macrophage lipid loading and plaque evolution [59]. Clinically, major cardiology guidelines continue to treat diabetes as a high-risk state requiring aggressive lipid management, reflecting the robust relationship between dyslipidemia-driven vascular injury and adverse outcomes [60].

Taken together, these observations indicate that diabetic dyslipidemia creates a vascular environment prone to endothelial activation and lipid retention. The specific molecular mechanisms underlying this lipotoxic stress are discussed below [10,57].

## 4. Lipid-Driven Injury Mechanisms

### 4.1. Lipotoxic Stress

Lipid abnormalities in diabetes extend beyond the kidney and exert direct molecular effects within the vasculature, contributing to macrovascular complications [61,62]. At the mechanistic level, hyperglycemia and excess lipid flux enhance oxidative stress and promote maladaptive lipoprotein modifications, increasing the burden of atherogenic and functionally impaired particles [56,61]. Modified LDL exhibits greater arterial wall retention and is more readily taken up by macrophages, facilitating foam cell formation and early plaque development [59,61]. In parallel, endothelial dysfunction—characterized by reduced nitric oxide bioavailability and upregulation of adhesion pathways—emerges as an early and organizing event in atherogenesis [57]. Collectively, these observations indicate that diabetic dyslipidemia exerts coordinated lipotoxic effects across both renal and vascular compartments, although the specific pattern of injury is shaped by local cellular and metabolic context [19,61].

### 4.2. Mitochondrial Dysfunction

Mitochondria are central to renal energy homeostasis, particularly in proximal tubular cells, which rely predominantly on FAO to sustain their high ATP demand [54,63,64]. In DKD, mitochondrial abnormalities are consistently reported, including impaired oxidative capacity, increased mitochondrial reactive oxygen species production, and disruption of mitochondrial dynamics and quality control mechanisms [63]. Defective FAO promotes the accumulation of lipid intermediates and lipid droplets, further compromising mitochondrial integrity and bioenergetic efficiency [31,54]. Rather than representing isolated events, these disturbances appear to be mechanistically interconnected: impaired mitochondrial function limits effective lipid utilization, whereas lipid overload aggravates mitochondrial stress and perpetuates maladaptive metabolic reprogramming in tubular cells [32,63].

### 4.3. Inflammation and Progressive Fibrosis

Lipid accumulation in DKD is closely associated with activation of inflammatory signaling pathways, as metabolic danger signals converge on innate immune programs to drive sterile inflammation within the renal parenchyma [65,66]. Activation of NF-κB–related transcriptional cascades together with inflammasome signaling amplifies the production of pro-inflammatory cytokines and sustains tissue injury [65,67,68].

Persistent inflammatory activation promotes fibroblast activation and extracellular matrix deposition through interconnected profibrotic networks, with TGF-β signaling serving as a central axis linking inflammatory stress to structural remodeling [54,55,62]. Over time, these processes culminate in tubulointerstitial fibrosis—a common pathological endpoint that strongly correlates with progressive decline in renal function [54,69].

### 4.4. Endothelial Dysfunction

In the vasculature, lipid abnormalities similarly drive endothelial activation and dysfunction, while diabetes-specific metabolic perturbations further accelerate atherosclerotic progression [61]. Oxidized and modified lipoproteins stimulate endothelial inflammatory signaling, enhance adhesion molecule expression, and impair nitric oxide–dependent pathways, thereby promoting leukocyte recruitment and vascular injury [56,57].

**Figure 2:**
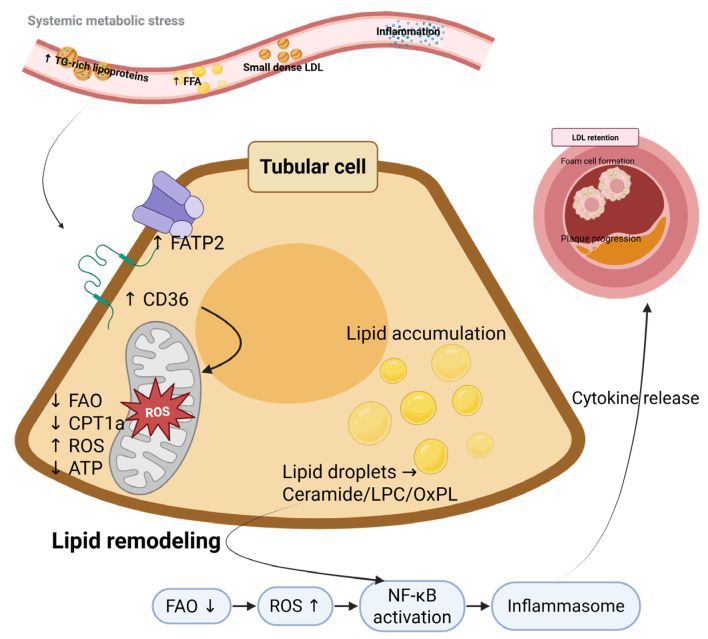
Integrated molecular pathways linking tubular lipid overload to inflammatory signaling and pro-atherogenic consequences. Systemic dyslipidemia increases renal lipid delivery, facilitating fatty acid uptake in tubular epithelial cells via upregulated CD36 and FATP2. Impaired mitochondrial FAO, accompanied by reduced CPT1A activity, promotes mitochondrial dysfunction, decreased ATP production, and excessive reactive oxygen species (ROS) generation. Elevated ROS levels and lipid remodeling processes lead to the accumulation of bioactive lipotoxic species, including ceramides, lysophosphatidylcholine (LPC), and oxidized phospholipids. These metabolic disturbances activate NF-κB signaling and inflammasome pathways, triggering inflammatory cytokine release. Tubular inflammatory signaling may propagate systemic vascular injury and contribute to atherosclerotic plaque progression. Created in BioRender. Zhang, C. (2026) https://BioRender.com/vd4jqeq (accessed on 9 March 2016).

In chronic kidney disease, HDL particles frequently become dysfunctional, exhibiting reduced anti-inflammatory activity and diminished cholesterol efflux capacity, changes that may further amplify vascular damage [70,71]. These alterations provide a mechanistic basis for the markedly elevated cardiovascular risk observed in individuals with DKD, consistent with contemporary cardio–renal risk frameworks [72,73]. Taken together, the evidence supports a unifying model in which tubular lipid overload drives a cascade of metabolic and inflammatory events. Impaired mitochondrial FAO, reduced CPT1A activity, and energy deficiency promote reactive oxygen species (ROS) overproduction and lipotoxic lipid remodeling, leading to activation of NF-κB signaling and inflammasome pathways. Rather than acting independently, these processes form a self-amplifying network that links metabolic inflexibility to inflammatory injury. Through cytokine release and pro-inflammatory signaling, tubular dysfunction may contribute to systemic vascular injury (Figure 2).

## 5. Kidney–Vessel Crosstalk

### 5.1. Systemic Metabolic Effects

DKD should be conceptualized as a manifestation of systemic metabolic failure rather than an isolated renal complication. It arises within a milieu of insulin resistance, chronic low-grade inflammation, and atherogenic dyslipidemia, all of which precede and amplify structural kidney injury [74,75]. In this context, declining renal function does not merely reflect metabolic dysfunction but further aggravates it, establishing a self-reinforcing cardio-renal metabolic loop.

As glomerular filtration declines, profound disturbances in lipoprotein metabolism emerge. Reduced lipoprotein lipase (LPL) activity, altered apolipoprotein composition (including apoCIII enrichment), and impaired hepatic clearance of triglyceride-rich lipoproteins promote accumulation of remnant particles and small dense LDL [10,70]. These particles are highly susceptible to oxidative modification and readily penetrate the vascular intima, thereby linking renal insufficiency to accelerated atherogenesis. Thus, the dyslipidemia of CKD is not simply quantitative but reflects qualitative shifts toward a more pro-inflammatory and pro-atherogenic lipoprotein profile.

HDL dysfunction represents a particularly important component of this metabolic disturbance. In CKD, HDL particles undergo structural remodeling and lipidomic reprogramming, resulting in impaired cholesterol efflux capacity and loss of antioxidative and anti-inflammatory properties [37]. Rather than serving as vasoprotective mediators, dysfunctional HDL may acquire pro-inflammatory characteristics, further compromising endothelial homeostasis [20]. Accordingly, lipid dysfunction in DKD extends beyond circulating concentrations and reflects a fundamental impairment of lipoprotein quality and function.

Systemic lipid dysregulation also exerts direct pathogenic effects within the kidney. Intrarenal lipid accumulation and lipid droplet formation are increasingly recognized as drivers of tubular injury and fibrogenesis [10,12]. Proximal tubular epithelial cells rely predominantly on fatty acid oxidation for ATP production; disruption of this pathway induces mitochondrial dysfunction, energetic stress, and activation of profibrotic transcriptional programs [19,54]. This metabolic shift from oxidative lipid utilization toward lipid storage constitutes a maladaptive energy reprogramming that links systemic dyslipidemia to structural kidney damage. Recent studies further demonstrate that mitochondrial oxidative injury can rewire tubular lipid metabolism, thereby accelerating fibrosis progression [26]. Collectively, these findings position lipid metabolic dysfunction as a mechanistic bridge between systemic metabolic disease and progressive renal injury.

### 5.2. Uremic Influences

Uremia acts as a biochemical amplifier that transforms renal insufficiency into systemic vascular pathology. As DKD progresses, accumulation of protein-bound uremic toxins—including indoxyl sulfate and p-cresyl sulfate—introduces additional drivers of endothelial dysfunction and vascular injury [76]. These toxins stimulate reactive oxygen species generation, activate NF-κB–dependent inflammatory pathways, and reduce nitric oxide bioavailability, thereby promoting vascular stiffness and atherosclerotic plaque progression. CKD is increasingly recognized as a state of dysregulated redox signaling characterized by excess oxidative stress and impaired antioxidant defenses [77]. Elevated reactive oxygen species disrupt mitochondrial function and further impair lipid metabolism, reinforcing metabolic stress at both systemic and renal levels. Oxidative modification of lipoproteins enhances their cytotoxicity toward endothelial and renal cells, intensifying vascular inflammation and tubular injury.

In parallel, uremia modifies lipoprotein composition and function. HDL particles in CKD exhibit lipidomic alterations that compromise reverse cholesterol transport and weaken vasoprotective signaling [37]. Within the kidney, lipid overload combined with mitochondrial stress induces maladaptive repair responses, promoting interstitial fibrosis and progressive nephron loss [19,26]. Therefore, the uremic milieu should not be viewed merely as a passive consequence of declining renal function, but rather as an active driver of metabolic, vascular, and fibrotic pathology.

### 5.3. Implications for Atherosclerosis

Atherosclerosis represents the clinical manifestation of kidney–vessel metabolic crosstalk. Large collaborative meta-analyses have demonstrated that even modest reductions in estimated glomerular filtration rate (eGFR) and the presence of albuminuria independently predict cardiovascular mortality, conferring a risk comparable to or exceeding that of diabetes alone [78,79]. These findings underscore that kidney dysfunction is not simply a marker of vascular disease but an independent determinant of cardiovascular risk.

Mechanistically, CKD promotes systemic inflammation, endothelial dysfunction, vascular calcification, and arterial stiffness—features consistent with accelerated vascular aging [20,77]. Reduced nitric oxide bioavailability, enhanced oxidative stress, and chronic inflammatory activation converge to accelerate atherogenesis. Conversely, vascular dysfunction may impair renal perfusion, exacerbate intraglomerular hypertension, and accelerate nephron loss, reinforcing a bidirectional pathogenic loop between kidney and vascular disease.

Therapeutic outcome trials further validate this interdependence. Sodium–glucose cotransporter 2 (SGLT2) inhibitors significantly reduce both renal and cardiovascular endpoints in patients with DKD and CKD [13,80,81], while nonsteroidal mineralocorticoid receptor antagonism with finerenone confers parallel cardiovascular and renal protection [82]. These data suggest that targeting shared metabolic, inflammatory, and hemodynamic pathways can interrupt the kidney–vessel axis. Interrupting this integrated cardio-renal-metabolic circuit may represent the most promising strategy to reduce the disproportionate cardiovascular burden associated with DKD.

### 5.4. Hypertension and Renal Hemodynamic Consequences

Hypertension may represent an additional hemodynamic manifestation of the fatty kidney phenotype. In diabetes, insulin resistance and increased proximal tubular glucose-sodium reabsorption reduce distal sodium delivery, blunt tubuloglomerular feedback, and promote glomerular hyperfiltration, sodium retention, and blood pressure elevation [83,84,85]. In parallel, ectopic lipid accumulation in and around the kidney may aggravate these abnormalities through local renal mechanisms. Rather than hyperglycaemia alone, fatty kidney may contribute to hypertension through local renal and neurohumoral mechanisms, including increased renal vascular resistance, impaired renal perfusion, and activation of sodium-retentive pathways [85,86,87]. Thus, hypertension in fatty kidney is likely driven by both metabolic transport abnormalities and lipid-associated renal hemodynamic stress, rather than by hyperglycemia alone.

These shared pathways also provide a mechanistic basis for the renal and cardiovascular benefits of SGLT2 inhibitors and nonsteroidal mineralocorticoid receptor antagonists. SGLT2 inhibitors reduce proximal tubular glucose-sodium reabsorption, restore tubuloglomerular feedback, promote natriuresis, and lower intraglomerular pressure, thereby interrupting a major pathway linking diabetes to hypertension and kidney injury [83,84]. Finerenone, by blocking mineralocorticoid receptor signaling, counteracts sodium-retentive, inflammatory, and profibrotic responses that further amplify renal vascular dysfunction and blood pressure-related injury [82,88]. Taken together, these observations support inclusion of hypertension within the broader kidney–vessel crosstalk framework of fatty kidney.

## 6. Conflicting Evidence

### 6.1. Pathogenic or Adaptive

Lipid accumulation is a consistent finding in CKD and DKD, yet its significance remains debated rather than settled [10,13]. In many studies, renal lipid deposition parallels inflammation, fibrosis, and declining function. However, this association does not necessarily establish lipid storage as purely pathogenic. Increasingly, it has been proposed that lipid accumulation—particularly in the form of lipid droplets—may initially represent a compensatory response to metabolic stress.

Lipid droplets are dynamic structures that respond to fluctuations in fatty acid supply rather than passive deposits of excess fat [89,90]. By converting free fatty acids into neutral triglycerides, they limit the availability of lipotoxic intermediates such as ceramides, diacylglycerols, and oxidized lipids. Under conditions of nutrient overload or impaired mitochondrial oxidation, this sequestration may reduce endoplasmic reticulum stress and oxidative damage, at least temporarily [89,90]. In several experimental settings, enhancing triglyceride storage attenuates acute lipid-induced injury, supporting the view that lipid droplet formation can serve a buffering function.

The difficulty arises when this buffering capacity is exceeded. If fatty acid influx surpasses the ability of cells to store or oxidize lipids safely, toxic lipid species accumulate [91,92]. Ceramides, in particular, are not simply by-products of lipid excess but active signaling molecules capable of promoting mitochondrial dysfunction, inflammasome activation, and cell death pathways [91,92,93]. In this context, lipid deposition may reflect metabolic inflexibility rather than protection [13,14]. Thus, lipid droplets may mark a transitional state: protective during early compensation but maladaptive when storage capacity is overwhelmed.

What remains unclear is whether lipid droplet accumulation directly contributes to injury or instead reflects upstream disturbances such as defective fatty acid oxidation. The balance between adaptive storage and maladaptive signaling likely varies across cell types and disease stages [10]. Without resolving this continuum, lipid deposition cannot be interpreted uniformly as either protective or injurious.

**Figure 3:**
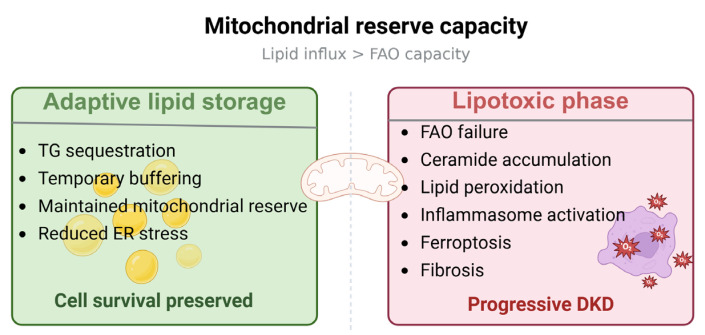
Mitochondrial reserve capacity and the transition from adaptive lipid storage to lipotoxic injury in DKD. In the setting of transient lipid excess, renal cells can buffer fatty acids through triglyceride sequestration and lipid droplet formation, thereby limiting lipotoxic stress and maintaining mitochondrial function. However, when lipid influx persistently exceeds fatty acid oxidation capacity, mitochondrial reserve becomes insufficient. This shift is associated with accumulation of bioactive lipid intermediates, lipid peroxidation, inflammatory activation, ferroptosis, and progressive fibrosis. Created in BioRender. Zhang, C. (2026) https://BioRender.com/krrw51i (accessed on 9 March 2016).

These considerations support the view that lipid deposition represents a stage-dependent phenomenon. Its impact may vary according to the balance between fatty acid delivery and mitochondrial oxidative capacity. When oxidative reserve is maintained, lipid sequestration may remain adaptive; when oxidative capacity is exceeded, lipid handling may instead contribute to inflammatory and fibrotic signaling (Figure 3).

### 6.2. Toxic Versus Neutral Lipids

The apparent contradictions in the literature are further complicated by the diversity of lipid species involved. Total lipid burden does not capture the functional heterogeneity of individual lipid subclasses. Neutral triglycerides stored within lipid droplets are generally considered less cytotoxic [89,90]. In contrast, saturated fatty acids and sphingolipid intermediates—particularly ceramides—have been implicated in apoptosis, insulin resistance, and inflammatory activation [91,92,93,94].

Ceramides illustrate how specific lipid species can exert biological effects disproportionate to their abundance. Changes in ceramide chain length or subcellular distribution may influence mitochondrial integrity and stress signaling [91,92]. This raises the possibility that lipid remodeling, rather than absolute lipid accumulation, may be more relevant to disease progression.

Lipidomic analyses have begun to reveal distinct lipid signatures associated with CKD and DKD [35,37]. Alterations in sphingolipids, oxidized phospholipids, and HDL-associated lipid fractions suggest that lipid dysregulation is selective rather than uniform. Moreover, lipid localization matters. Lipids incorporated into membranes, stored in droplets, or accumulating within mitochondria may exert different biological effects [10,95]. Studies that quantify total renal lipid content without resolving species or compartments may therefore overlook functionally important differences.

At present, it remains uncertain which lipid species are true drivers of injury and which reflect downstream metabolic disturbance. This uncertainty cautions against broadly targeting “renal lipid accumulation” without distinguishing between neutral storage lipids and bioactive mediators [13,14].

### 6.3. Why Studies Disagree

Discrepancies across studies likely reflect both biological variability and technical differences. Experimental models vary widely in duration and type of metabolic stress, from acute ischemic injury to chronic hyperglycemia and genetic alterations in lipid metabolism [14,96]. These conditions are not interchangeable, yet findings from them are often discussed collectively under the concept of lipotoxicity.

Cell type also matters. Proximal tubular epithelial cells, which rely heavily on fatty acid oxidation, may be particularly sensitive to disruptions in lipid handling. Podocytes and infiltrating immune cells, however, may respond differently to the same lipid environment. In some models, lipid droplet accumulation tracks with fibrosis severity; in others, it appears without immediate structural deterioration [10,13]. Such variability suggests that lipid deposition may reflect distinct metabolic adaptations rather than a single pathogenic pathway.

Methodological differences further complicate interpretation. Histological staining predominantly detects neutral lipid droplets, whereas lipidomics identifies specific lipid subclasses, including signaling-active species [35,37]. As a result, two studies reporting “increased lipid accumulation” may in fact be describing different biochemical phenomena. Without distinguishing storage lipids from toxic intermediates, conclusions may appear inconsistent.

Finally, lipid metabolism is tightly linked to redox signaling and mitochondrial function [77,95]. Because these processes are dynamic, single-time-point measurements may capture transient adaptation or late-stage injury without clarifying sequence. It remains unresolved whether lipid accumulation precedes renal injury, follows mitochondrial dysfunction, or participates in a bidirectional feedback loop [14,94]. Addressing these questions will require longitudinal and cell-type–specific analyses that integrate lipidomic and functional data [13,35].

## 7. Therapeutic Implications

### 7.1. Targeting Lipid Metabolism

Lipid dysmetabolism is increasingly viewed as a causal component of diabetic kidney disease rather than a secondary epiphenomenon [10]. In diabetic tissues, excessive fatty acid uptake, impaired β-oxidation, and aberrant lipid storage converge to produce lipotoxic stress [10,97]. This shifts the therapeutic focus from plasma lipid levels to intracellular lipid flux [98]. CD36 sits at the center of this axis. It regulates long-chain fatty acid uptake and links extracellular lipid availability to intracellular metabolic programming [97,99]. CD36 trafficking and activity are tightly controlled by post-translational mechanisms, including palmitoylation, and directly influence oxidative metabolism and inflammatory signaling [97,99,100]. Beyond uptake, CD36 intersects with autophagy and lipid droplet turnover, integrating lipid handling with stress responses [101]. In diabetic kidney and vascular cells, sustained CD36 activation amplifies oxidative injury and fibrotic remodeling [10,99].

Defective fatty acid oxidation in tubular epithelial cells represents a parallel and equally important defect in DKD [10,19,54]. Pharmacologic activation of PPARα or dual PPARα/δ signaling restores mitochondrial function, reduces lipid droplet accumulation, and mitigates tubular injury in experimental models [102,103]. Selective PPARα modulators (SPPARMα) aim to retain these metabolic effects while minimizing off-target toxicity [104]. Epigenetic regulation further stabilizes PPAR-dependent metabolic programs in diabetic tissues [105]. Importantly, therapeutic strategies should not indiscriminately eliminate lipid storage. Lipid droplets can buffer toxic intermediates under stress [10,98]. The goal is restoration of balanced lipid flux—coordinating uptake, oxidation, and storage—rather than simple lipid depletion [98].

Conventional lipid-lowering therapies such as statins and fibrates remain important for improving circulating dyslipidaemia and reducing cardiovascular risk [49,60]. However, within the fatty kidney framework, their direct effects on intrarenal lipid trafficking, glomerular lipid handling, proximal tubular fatty acid oxidation defects, and bioactive lipotoxic signaling are likely limited [10,19]. Emerging therapies are therefore of particular interest when they more directly modulate the abnormal lipid metabolism of the kidney itself rather than primarily correcting systemic lipid concentrations.

Several mechanism-based approaches may complement conventional lipid-lowering. First, the gut–kidney axis has emerged as an upstream regulator of renal lipid dysmetabolism. Recent reviews in DKD suggest that gut microbiota dysbiosis may aggravate kidney injury through altered short-chain fatty acid production, impaired intestinal barrier function, lipopolysaccharide signaling, uremic toxin generation, and broader host metabolic reprogramming, thereby indirectly promoting intrarenal lipid accumulation and inflammatory injury [106,107]. This concept is supported by mechanistic studies showing that aberrant gut microbiota alter host metabolome in renal failure and that gut bacteria-derived metabolites can attenuate experimental kidney injury [108,109]. Human evidence remains limited, but the SYNERGY randomized trial demonstrated that synbiotics modified the stool microbiome and reduced serum p-cresyl sulfate in CKD, supporting the clinical plausibility of gut microbiota-targeted intervention [110].

Second, therapies that more directly target renal lipid handling are increasingly relevant. PCSK9 is already implicated in glomerular lipid accumulation and renal injury in DKD [29], suggesting that PCSK9-targeted therapy may act not only through systemic lipid lowering but also through direct effects on glomerular lipid homeostasis. In parallel, PP2A-targeted modulation has emerged as a mechanistically relevant strategy, as arctigenin attenuated DKD through activation of PP2A in podocytes and suppression of inflammatory signaling, linking PP2A-related signaling to the correction of maladaptive injury responses in the fatty kidney [111]. In addition, Keluoxin reduced renal ectopic lipid deposition by inhibiting lipid synthesis, promoting fatty acid oxidation, and modulating the AMPK/NF-κB axis [112], whereas liraglutide alleviated renal tubular ectopic lipid deposition by inhibiting lipid synthesis and promoting lipolysis [113]. Recent work further suggests that FGF1 may reduce renal lipid accumulation while improving inflammation, fibrosis, and albuminuria in diabetic kidney disease [114]. 

Taken together, these emerging approaches differ from traditional statin- or fibrate-based treatment in that they may act more directly on the abnormal lipid metabolism of the fatty kidney itself, including glomerular lipid accumulation, proximal tubular lipid uptake, mitochondrial substrate utilization, and lipotoxic signaling. Nevertheless, most of these strategies remain preclinical or early translational, and further studies are needed to determine whether targeting intrarenal lipid dysmetabolism will yield durable renal benefit in patients with DKD.

### 7.2. Cardiovascular Effects

Cardiovascular outcome trials with SGLT2 inhibitors and Glucagon-Like Peptide-1 (GLP-1) receptor agonists provide the strongest clinical evidence that metabolic reprogramming translates into organ protection [115,116,117]. Both drug classes consistently reduce major adverse cardiovascular events and slow kidney disease progression in type 2 diabetes [115,116,118,119]. These benefits persist across different levels of baseline renal function [118,119].

Neither class was developed as a lipid-targeted therapy, yet both influence systemic substrate utilization. SGLT2 inhibitors shift metabolism toward enhanced lipid oxidation and ketone utilization, improving mitochondrial efficiency and reducing oxidative stress [120]. GLP-1 receptor agonists reduce weight, improve insulin sensitivity, and attenuate inflammatory signaling [115,116]. Comparative effectiveness analyses confirm reductions in kidney failure and mortality among patients with diabetes and CKD [118,119]. Combination therapy is therefore mechanistically rational, given its complementary metabolic effects [121]. Together, these data support a unified cardiorenal paradigm in which systemic metabolic remodeling indirectly normalizes lipid handling in both renal and vascular tissues [116,117,120].

### 7.3. Open Challenges

Key mechanistic gaps remain. It is unclear which lipid species drive pathology and which serve adaptive roles [10,98]. Total lipid accumulation may obscure the contribution of bioactive intermediates that directly impair mitochondrial function [10]. Moreover, lipid handling is highly cell-specific. Tubular epithelial cells, podocytes, endothelial cells, and cardiomyocytes operate under distinct metabolic constraints [10,97]. Fatty acid uptake is regulated at multiple levels—transcriptional, post-translational, and trafficking-dependent—making uniform targeting unlikely to succeed [98,100].

Timing is also critical. Early metabolic derangements may be reversible, whereas established fibrosis limits responsiveness [10]. Emerging spatial lipidomics and single-cell approaches offer an opportunity to define pathogenic lipid signatures with cellular precision [10]. Ultimately, effective therapy will require restoration of metabolic flexibility within the cardiorenal axis, not simply suppression of lipid burden [10,120].

## 8. Conclusions

The concept of “fatty kidney” provides a mechanistic framework that links lipid dysregulation to mitochondrial dysfunction, inflammation, and progressive structural injury in diabetic kidney disease. Rather than representing a passive epiphenomenon of systemic dyslipidemia, renal lipid accumulation reflects coordinated disturbances in lipid uptake, intracellular processing, and oxidative metabolism. Increasing evidence indicates that altered lipid homeostasis within glomerular and tubular compartments contributes directly to disease progression [96].

Among renal cell types, proximal tubular epithelial cells are particularly susceptible to metabolic imbalance because of their high reliance on mitochondrial fatty acid oxidation for ATP production. When oxidative capacity is impaired, fatty acids cannot be efficiently metabolized, resulting in lipid droplet accumulation and generation of bioactive lipid intermediates. Recent work demonstrates that mitochondrial metabolic reprogramming is a defining feature of DKD and that disrupted mitochondrial function reshapes cellular lipid utilization pathways [27]. Complementary mechanistic studies further show that mitochondrial oxidative damage can actively reprogram lipid metabolism in tubular epithelial cells, reinforcing a feed-forward loop between energetic stress and lipid dysregulation [26]. Together, these findings support the view that fatty kidney reflects a breakdown of metabolic flexibility rather than simple lipid overload.

Importantly, lipid deposition should not be interpreted uniformly as pathogenic. Lipid droplets are dynamic organelles that may initially buffer excess fatty acids and protect against acute lipotoxic stress. However, when lipid influx persistently exceeds oxidative and storage capacity, accumulation of toxic intermediates—including peroxidized lipids—can amplify oxidative stress, inflammatory signaling, and profibrotic pathways. Emerging evidence highlights lipid peroxidation as a critical mediator linking metabolic imbalance to renal cell injury in DKD [122]. Thus, fatty kidney likely represents a continuum, ranging from adaptive sequestration to maladaptive lipotoxic signaling depending on disease stage and mitochondrial reserve.

The fatty kidney phenotype also develops within a broader systemic metabolic environment. Diabetic dyslipidemia promotes parallel lipid disturbances in vascular tissues, contributing to endothelial dysfunction and atherosclerotic progression. While renal tubular cells respond primarily through energetic stress and fibrotic remodeling, vascular cells undergo lipid retention and inflammatory activation. This shared metabolic background underscores the interconnected nature of kidney and vascular injury in diabetes. Viewing fatty kidney within a cardio–renal–metabolic framework helps explain why interventions that improve systemic metabolic efficiency often yield simultaneous renal and cardiovascular benefits.

Therapeutically, these insights shift attention from static measurements of circulating lipids toward dynamic regulation of intracellular lipid flux and mitochondrial competence. Restoration of balanced lipid handling—through modulation of fatty acid uptake, enhancement of FAO, or limitation of lipid peroxidation—may represent a more precise strategy than indiscriminate lipid depletion. As mechanistic understanding advances, integrating lipidomics, mitochondrial biology, and cell-specific metabolic profiling will be essential to distinguish adaptive from pathogenic lipid responses.

In summary, the fatty kidney should not be regarded merely as a descriptive term for renal lipid deposition, nor should it be considered interchangeable with metabolic inflexibility. Rather, it is best understood as a phenotype of ectopic renal lipid accumulation, often driven by impaired renal lipid handling and metabolic inflexibility, in which persistent lipid influx overwhelms mitochondrial oxidative capacity and promotes inflammatory and fibrotic remodeling.

## Data Availability

No new data were created or analyzed in this study.
